# Tasty Bits a Dutch Treat

**DOI:** 10.3201/eid1601.AC1601

**Published:** 2010-01

**Authors:** Polyxeni Potter

**Affiliations:** Author affiliation: Centers for Disease Control and Prevention, Atlanta, Georgia, USA

**Keywords:** Pieter Claesz, still life painting, food safety, Dutch Golden Age, Still Life with Turkey Pie, art science connection, emerging infectious diseases, art and medicine, about the cover

**Figure:**
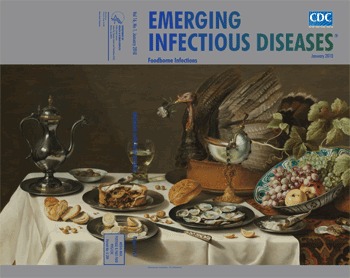
**Pieter Claesz (1597–1660) Still Life with Turkey Pie (1627).** Oil on wood panel (75 cm × 132 cm) Rijksmuseum, Amsterdam, the Netherlands

“The old Dutch whalers of two or three centuries ago,” wrote Herman Melville in Moby-Dick, “were high livers.” The author arrived at this conclusion by transcribing what a ship with a crew of 100 carried for each month at sea in 1636 into what a fleet of 180 Dutch whalers might carry: “400,000 lbs of beef, 60,000 lbs Friesland pork, 150,000 lbs of stock fish, 550,000 lbs biscuit, 72,000 lbs soft bread, 2,800 firkins of butter … 10,800 barrels of beer.” This tale of whale fishery is not unlike other accounts of legendary feasting in the Netherlands during the 17th century, the Dutch Golden Age.

Though fishing was a substantial part of the Dutch economy, it was trade with countries near and afar that converted tiny Holland into a vast empire. Described as “the best fed population in Europe,” the Dutch enjoyed a high standard of living. Food that was not grown domestically was imported from all over the world, and even the poor “were supplied with fare meant to approximate to the diet of the more fortunate.” Though generally avert to excess, Netherlanders celebrated the birth of a child, the New Year, the purchase of a house, the departure or return of a family member, the wedding or funeral of a friend with a sumptuous feast, tinged with the tastes of India and the Spice Islands and washed down with the best wines from Spain, Italy, and France.

Commercial prosperity created a large affluent society, demand for exotic goods and luxuries, and thriving art markets in Delft, Haarlem, The Hague and other cities. Though shaken by the iconoclastic fervor of Protestant Reformation as it spread throughout northern Europe and the loss of patronage by the Catholic Church, the art scene remained competitive to accommodate burghers seeking artwork for their large homes. Artists became specialized to meet the collectors’ interests in secular subjects that showcased their newly acquired wealth through genre scenes and still life paintings.

Still life paintings, the “foot soldiers in the army of art,” as they have been called for their modest ranking, found a niche as an independent form beginning in the 16th century at the same time in Italy, Spain, and northern Europe. While natural philosophers turned to investigation as a tool for learning and in Delft, Antonie van Leeuwenhoek was seeing “with great wonder…many very little living animalcules” with his microscope, artists shifted their focus from mythologic subjects to landscapes, animals, and plants. Art captured the bounty of God’s creation, providing not only an affirmation of wealth but also an opportunity for symbolism and moral lessons, in line with Calvinist doctrine. In the midst of abundance, the Dutch feared becoming corrupted by it, so their art often contained proverbs and other warnings against frivolity and excess.

Still lifes encompassed all sorts of inanimate objects and even some small animals or insects. Some, called banquet pieces, were lavish arrangements of food and table settings. Others portrayed breakfast offerings of herring, bread, beer, or wine. Fine artists of the period painted these, among them Pieter Claesz, a master of visual feasts. Very little is known about Claesz’ life. A native of Berchem, a village near Antwerp, he likely began his studies there but later moved to the larger art community in Haarlem, where he was noticed for his innovative style. He was very prolific. His work was widely imitated, copied, and reproduced during his lifetime. His son, Nicolaes Pietersz Berchem became an important landscape artist.

Claesz painted scenes with bowls, glasses, and other objects luxuriously appointed and filled with spices, fruit, and allusions to the fleeting nature of earthly pleasures and the vanity of life. He pioneered a nearly monochromatic palette with subdued hues and achieved remarkable naturalism by focusing on how light affects a scene and how objects are perceived against each other and as reflections on highly polished surfaces. Along with still life greats Willem Heda and Clara Peeters, he created the subtle and refined banquet style identified with the Haarlem art scene.

In Still Life with Turkey Pie, on this month’s cover, the muted colors alone betray Claesz’ genius. They allow the light to reflect off the tumbler of white wine, the nautilus shell goblet, the plates, even the glistening olives and grapes. A glimpse of the room can be had on the pewter pitcher. The turkey, having supplied the namesake feast, also donated its light-streaked feathers and beak as decorative headdress for the raised pie.

The composition engages the senses and turns a still life into a dynamic one, a banquet in progress, even without humans. We are invited to sample. A plate and knife balance off the edge of the table within reach, a serving spoon rests atop the half-eaten mince pie. The lemon is half peeled. The tablecloth, live with folds and shadows of objects and set against a rug, bleeds to the edge. A Delft bowl rests invitingly at an angle filled with fruit and crowned with leaves and twigs. The formality is broken by bread placed directly on the cloth, along with a star fruit and nuts cracked and strewn between the plates along with slices of lemon and empty seashells.

Mince pie, flavored with currants and spices, was a treat for special occasions, as were imported lemons and olives. Salt and the pepper poured out of a rolled paper cone made a fitting complement to the luxury of oysters and white bread. The fruit was meant to whet the senses and stimulate the appetite as were the shellfish, a didactic treat, what with their empty shells hinting at the ephemeral.

The abundance and variety of food so eloquently celebrated in art of the Golden Age did not escape the attention of those responsible for its safe distribution and use. The quality of perishables was monitored, and government regulations banned the sale of rotting vegetables and fruits. Bakers were required to wash their feet with hot water and nontoxic soap before using them to knead tough rye dough. Food preservation techniques, among them smoking, drying, and pickling, were widely practiced. And against a common misconception, spices were used to enhance the flavor of food, not to cover its taste.

Despite van Leeuwenhoek’s observation in the mouth of an old man of “an unbelievably great company of living animalcules,” or bacteria, no connection had yet been made between them, or the other microorganisms he discovered, and human disease. Yet, food, then as now, was linked to disease, often unfairly. In 1655, blue plums and black cherries, blamed for the plague because of their close resemblance to buboes, were temporarily banned, and pineapple was thought by some to cause “gastric ailments from the Orient.”

Centuries later, the abundance of food in some parts of the world rivals that of the Dutch Golden Age. But neither the distribution nor the safety of the global food supply has become foolproof. While microbiology has revolutionized the way we perceive food safety, movement of people and goods has compromised it. Now as in the 17th century, consumers in affluent societies sit at sumptuous banquets to a mythical array of goods, bar none. Yet, sweet or sour, rare or local, organic, bioengineered, opulent, or simply served, food carries warnings, no longer about frivolity and excess but about disease risk. When van Leeuwenhoek’s animalcules are pathogenic, each meal becomes a gamble. Infection, the great equalizer, has turned banquet to Dutch treat.
